# Supplementary material for: Sucrose-driven carbon redox rebalancing eliminates the Crabtree effect and boosts energy metabolism in yeast
Source: Nat Commun. 2025 Jun 5;16:5211. doi: 10.1038/s41467-025-60578-8 (PMC12141580; doi:10.1038/s41467-025-60578-8)
Supplement: Supplementary file 1 — Supplementary Information [file 41467_2025_60578_MOESM1_ESM.pdf]

Sucrose-driven carbon redox rebalancing eliminates the Crabtree effect and boosts energy metabolism in yeast

Xiao *et al.*

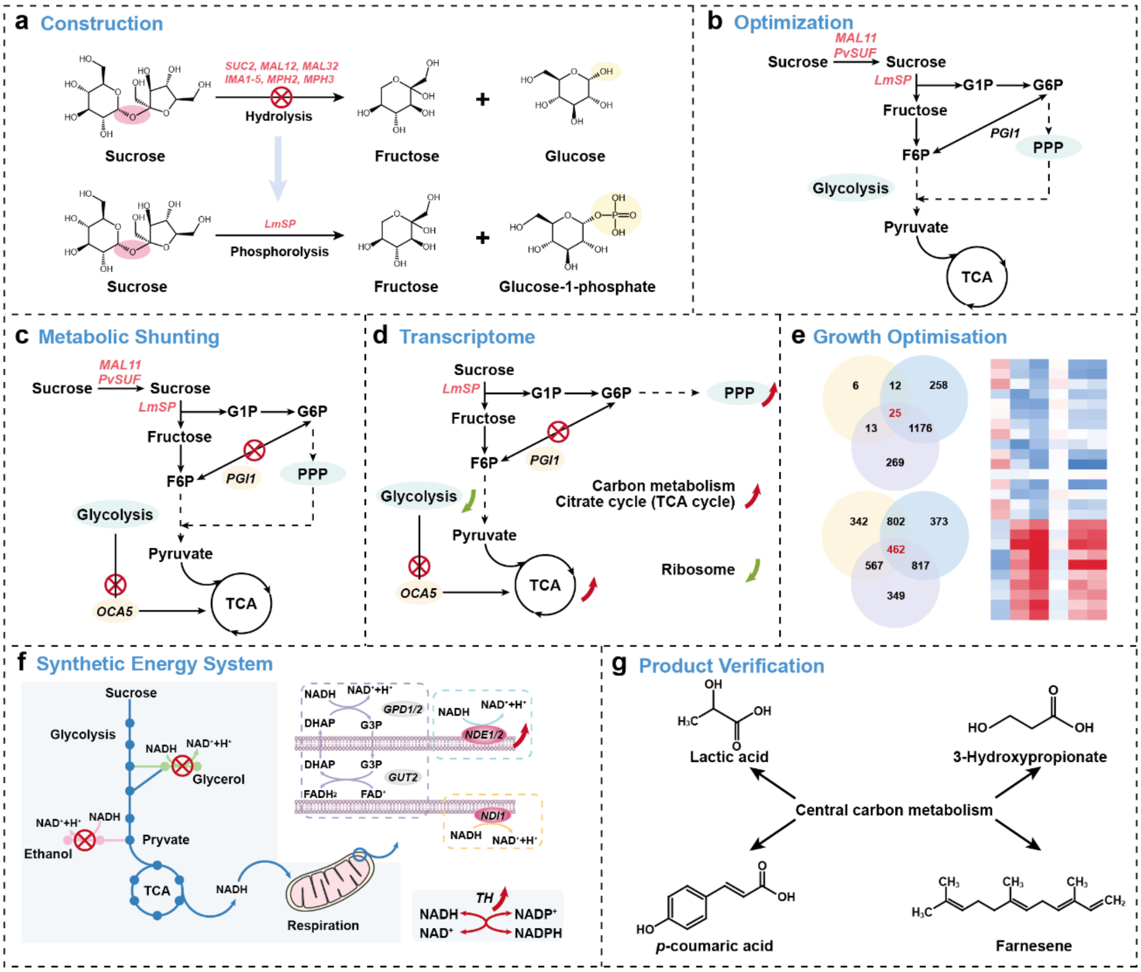

Supplementary Fig. 1. Construction process of the Crabtree-negative strains with synthetic energy system based on sucrose phosphorolysis.

a. Substitute the endogenous hydrolysis pathway with the sucrose phosphorolysis pathway. SUC2, extracellular sucrose hydrolase; MAL12, MAL32, α-D-glucosidase; IMA1-5, isomaltase; MPH2, MPH3, α-glucoside permease; MAL11, α-glucoside transporter; LmSP, sucrose phosphorylase. **b.** Optimize transport proteins to reduce the energy required for sucrose transport. PvSUF, sucrose facilitator. **c.** Reprogram central metabolism based on sucrose phosphorylation to transform yeast to the Crabtree-negative strain. **d.** Conduct transcriptomic analysis to verify the metabolic shift towards a Crabtree-negative state. **e.** Identify key genes involved in regulating cell growth. **f.** Develop a synthetic energy system to boost cellular ATP metabolism. NDE1, NDE2, Mitochondrial external NADH dehydrogenase; NDI1, internal NADH dehydrogenase; TH, transhydrogenase; GPD1, GPD2, NAD-dependent glycerol 3-phosphate dehydrogenase; GUT2, glycerol kinase. **g.** The Crabtree-negative yeast with a synthetic energy system applied to the synthesis of various compounds. Red crosses indicated knockout genes. Red arrows indicate upregulated pathways or genes and green arrows indicate downregulated pathways.

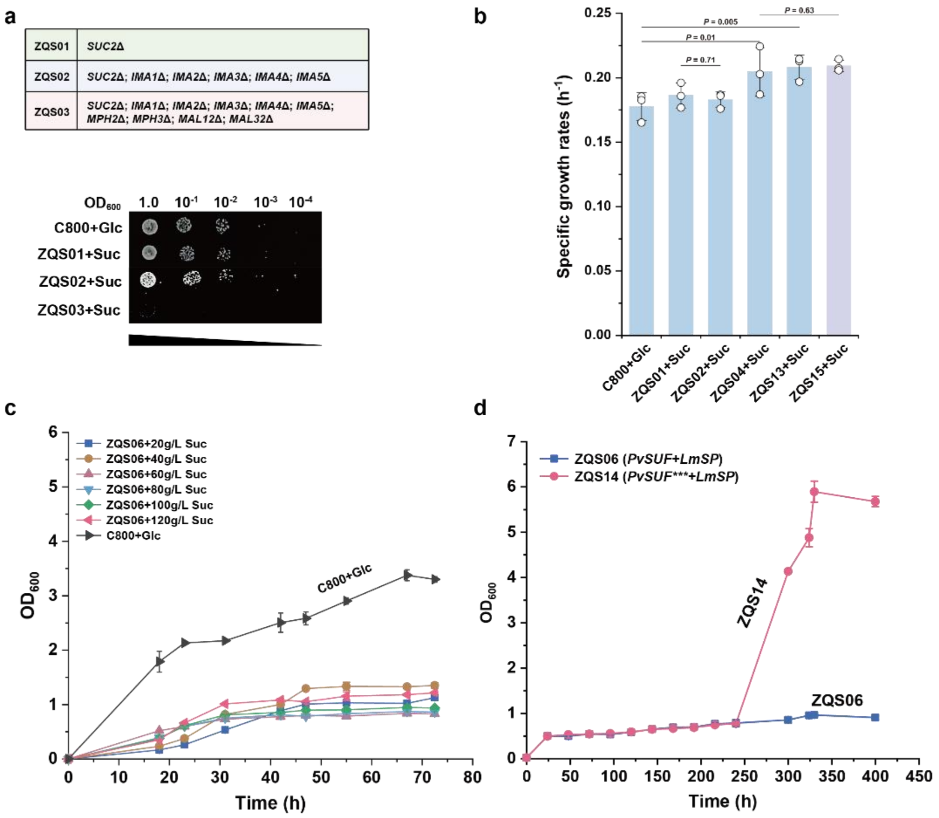

Supplementary Fig. 2. Evaluation of the impact of carbon source concentration and transport proteins on the sucrose phosphorylation pathway.

a. Spotting experiments for sucrose hydrolysis-related gene knockouts. C800 serves as the control strain, while ZQS01-ZQS03 represent strains with varying deletions of sucrose hydrolase genes. ZQS03 lacks *SUC2*, *MAL12*, *MAL32*, *IMA1-5*, *MPH2*, and *MPH3*. **b.** The specific growth rate of strains ZQS01-ZQS15. **c.** The growth of ZQS06 at different sucrose concentrations (20-120 g/L). ZQS06 is genotyped as Δ*MAL11*, *PvSUF* + *LmSP*. **d.** Comparison of growth between strains containing PvSUF-modified proteins and unmodified strains. The control strain, ZQS06 (Δ*MAL11*, *PvSUF* + *LmSP*), contains unmodified *PvSUF*, while ZQS14 harbors the modified $PvSUF^{I209F\ C265F\ G326C}$. All data are presented as mean ± SD of biological triplicates. Statistical analysis was conducted using Student's *t*-test (two-tailed; sample size, *n* = 3). Source data are provided as a Source Data file.

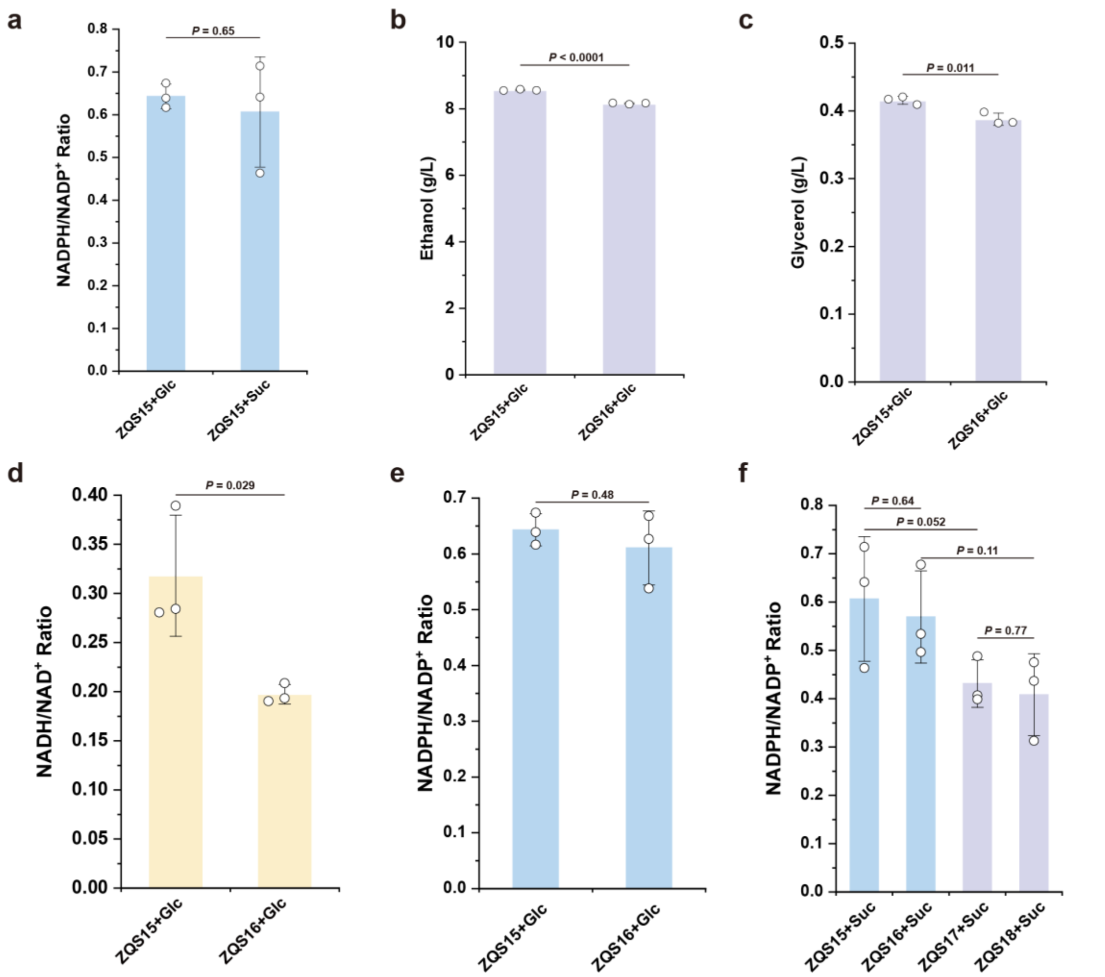

Supplementary Fig. 3. Validation of central carbon metabolism-related parameters in strains ZQS15-ZQS18.

a. $NADPH/NADP^+$ ratio of strain ZQS15 under glucose metabolism and sucrose phosphorolysis metabolic modes. **b.** Ethanol accumulation in ZQS16 during glucose metabolism. **c.** Glycerol accumulation in ZQS16 during glucose metabolism. **d.** Comparison of the $NADH/NAD^+$ ratio between ZQS16 and ZQS15 during glucose metabolism. **e.** $NADPH/NADP^+$ ratio of strain ZQS16 under sucrose phosphorolysis metabolic mode. **f.** $NADPH/NADP^+$ ratio in strains ZQS17 and ZQS18. All data are presented as mean ± SD of biological triplicates. Statistical analysis was conducted using Student's *t*-test (two-tailed; sample size, $n = 3$). Source data are provided as a Source Data file.

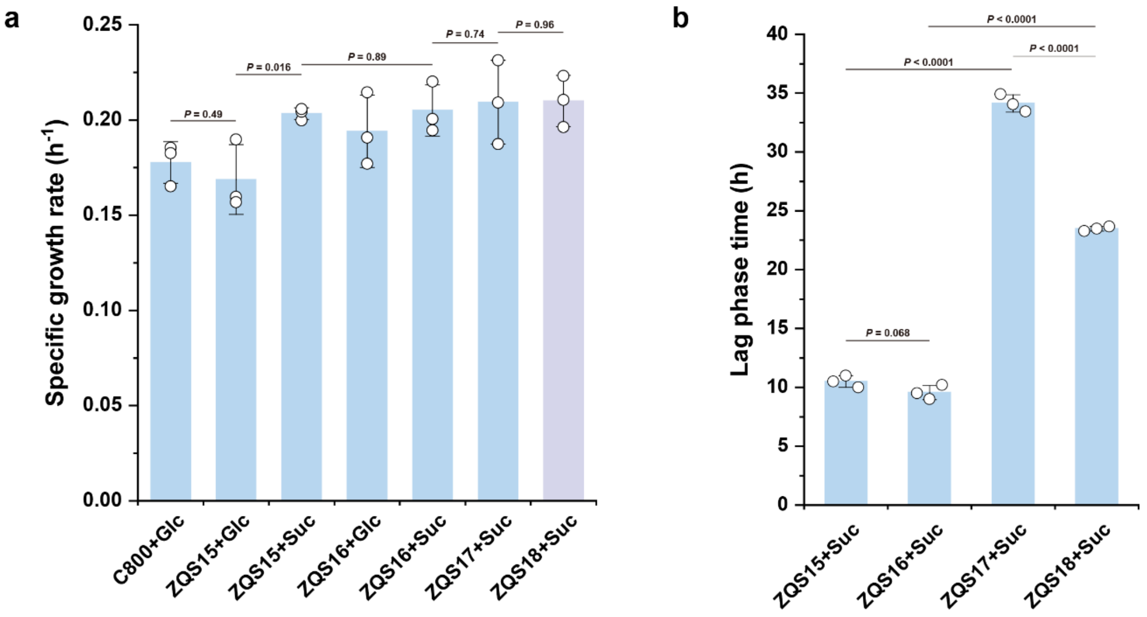

Supplementary Fig. 4. The specific growth rate and lag phase time of strains ZQS15-ZQS18.

a. The specific growth rates of ZQS15–ZQS18. C800 + Glc served as the control, representing the specific growth rate of strain C800 cultured in YPD medium with glucose as the carbon source. **b.** The lag phase time of ZQS15–ZQS18. All data are presented as mean ± SD of biological triplicates. Statistical analysis was conducted using Student's *t*-test (two-tailed; sample size, *n* = 3). Source data are provided as a Source Data file.

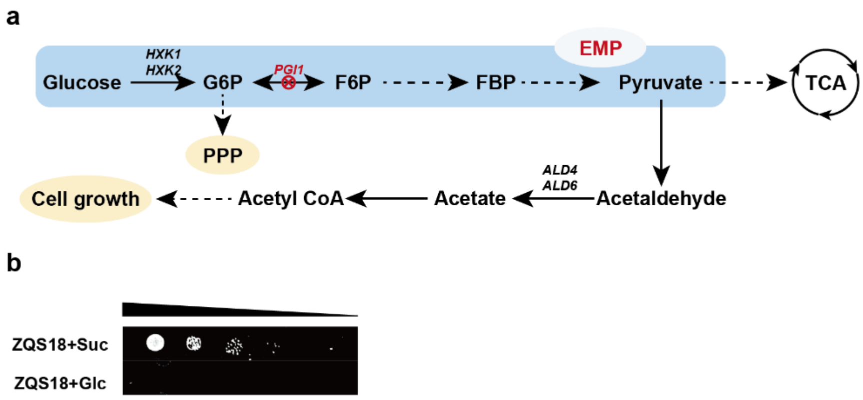

Supplementary Fig. 5. The knockout of *PGI1* shows a growth defect on glucose medium.

a. Glucose metabolism pathway in the *PGI1* knockout strain. The knockout of PGI1 disrupts the EMP pathway, impairing normal cellular metabolism. **b.** Spotting assay for the *PGI1* knockout strain. The *PGI1*Δ strain is unable to grow on glucose medium.

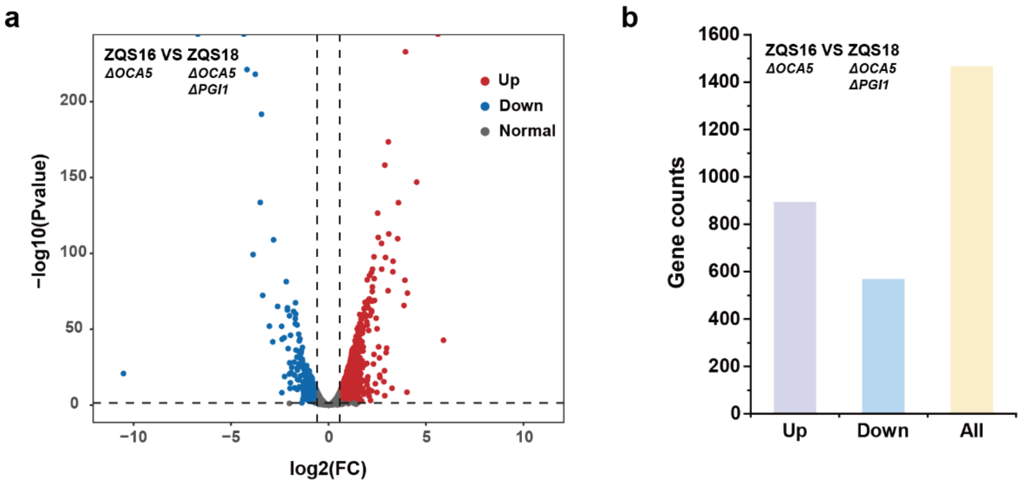

Supplementary Fig. 6. The differences in gene expression between the Crabtree-negative ZQS18 and Crabtree-positive strains ZQS16.

a. Volcano plot showing gene expression differences between ZQS18 and ZQS16. Significantly upregulated genes are highlighted in red, and significantly downregulated genes are highlighted in blue. **b.** Count of differentially expressed genes in the comparison between ZQS18 and ZQS16.

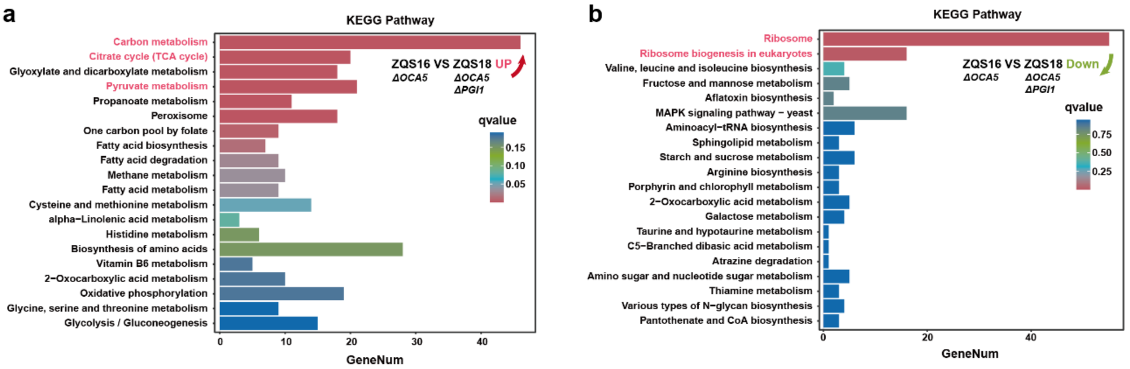

Supplementary Fig. 7. KEGG enrichment of genes in ZQS18 (Crabtree-negative) compared with ZQS16 (Crabtree-positive).

a. KEGG enrichment analysis of the upregulated DEGs in ZQS18 compared to ZQS16. The horizontal axis shows the number of enriched genes, while the vertical axis lists the pathway entries. Bar color represents the *q*-value, with smaller *q*-value indicating more significant gene enrichment in the pathway. **b.** KEGG enrichment analysis of downregulated DEGs in ZQS18 compared to ZQS16.

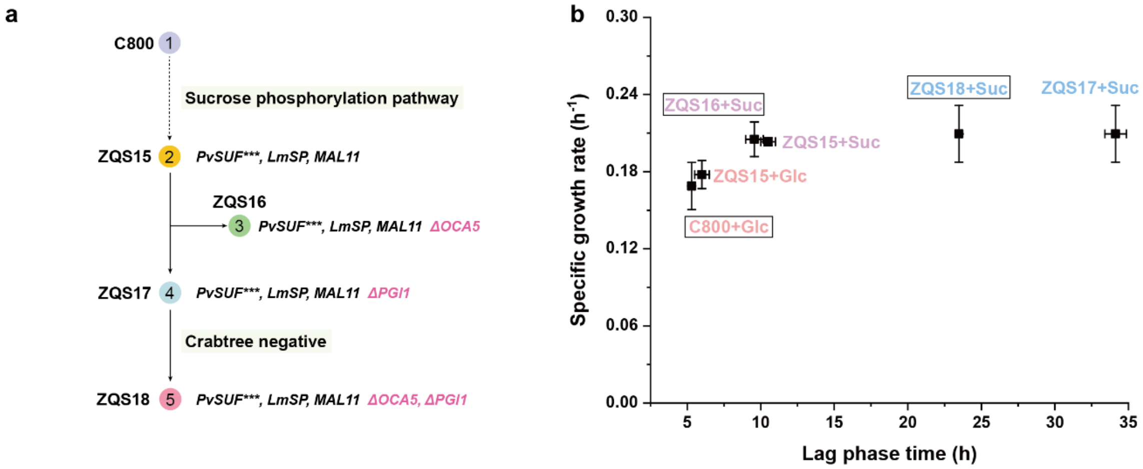

Supplementary Fig. 8. Comparison of lag phase time and specific growth rate among different strains.

a. Diagram of strain construction. C800 is the control strain. ZQS15 and ZQS16 are Crabtree-positive strains, while ZQS17 and ZQS18 are Crabtree-negative strains for sucrose metabolism. **b.** Font color indicates different growth conditions: pink for control strains under glucose metabolism, purple for strains under sucrose phosphate metabolism, and blue for *PGI1*Δ strains. Points closer to the top left of the coordinate axis represent better cell growth, while those near the bottom right indicate slower growth. All data are presented as mean ± SD of biological triplicates. Source data are provided as a Source Data file.

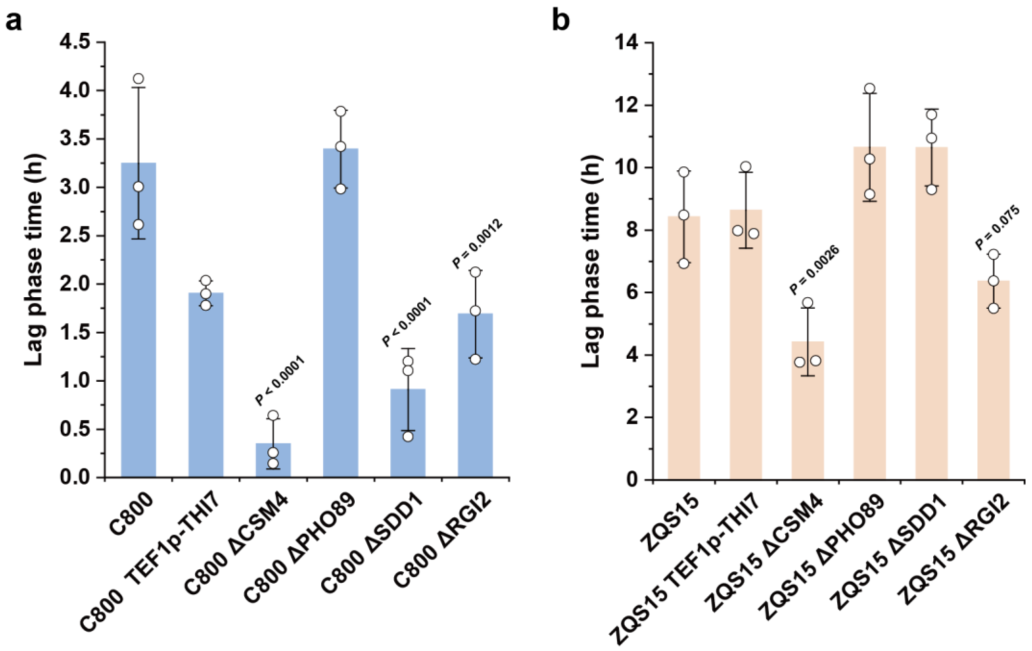

Supplementary Fig. 9. Validating the function of growth-regulating genes in glucose metabolism mode and sucrose phosphorolysis metabolism mode.

a. Effects of various genetic manipulations on the lag phase of strain C800, with C800 serving as the control strain. **b.** Effects of various genetic manipulations on the lag phase of strain ZQS15, with ZQS15 serving as the control strain. All data are presented as mean ± SD of biological triplicates. Statistical analysis was conducted using Student's *t*-test (two-tailed; sample size, $n = 3$). Source data are provided as a Source Data file.

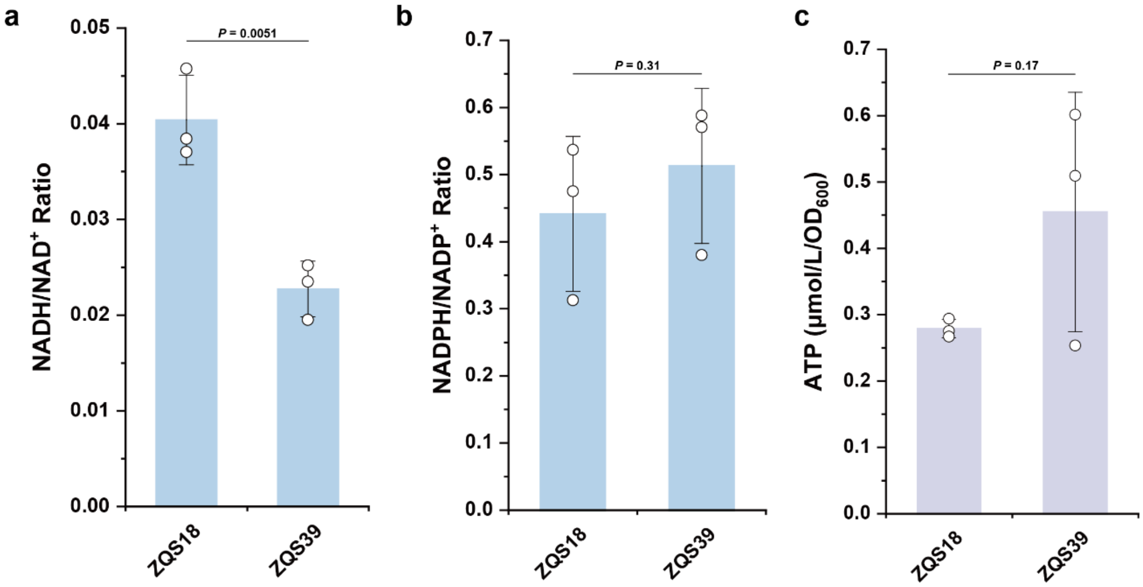

Supplementary Fig. 10. Comparison of central carbon metabolism-related parameters between ZQS39 and ZQS18.

ZQS18 is the control strain, and ZQS39 represents the *RGI2* knockout strain. **a.** Comparison of the $NADH/NAD^+$ ratio between ZQS39 and ZQS18. **b.** Comparison of the $NADPH/NADP^+$ ratio between ZQS39 and ZQS18. **c.** Comparison of ATP content between ZQS39 and ZQS18. All data are presented as mean ± SD of biological triplicates. Statistical analysis was conducted using Student's *t*-test (two-tailed; sample size, $n = 3$). Source data are provided as a Source Data file.

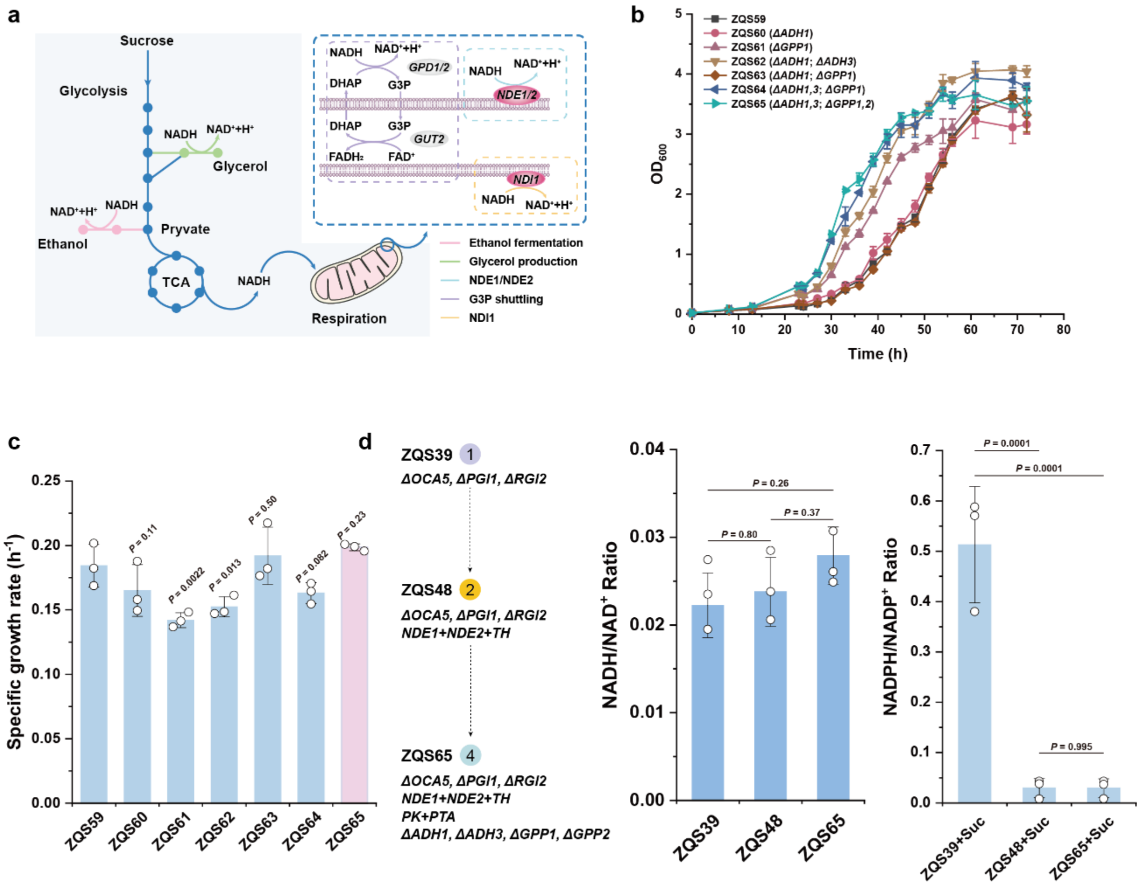

Supplementary Fig. 11. Impact of constructing and optimizing synthetic energy systems on cell growth, NADH/NAD$^+$ and NADPH/NADP$^+$ ratio.

a. Pathways of NADH reoxidation in yeast. There are a total of five pathways for NADH reoxidation in yeast, each represented by a different color indicating distinct oxidation methods. The pink pathway represents ethanol fermentation, green pathway represents glycerol production, blue pathway illustrates mitochondrial outer membrane NADH dehydrogenases (*NDE1*/*NDE2*) channeling NADH into the respiratory chain, purple pathway depicts glycerol-3-phosphate shuttling, and yellow pathway shows the role of mitochondrial NADH by the inner membrane NADH dehydrogenase NDI1 in transferring NADH into the respiratory chain. **b.** Growth curves of strains ZQS59-ZQS65. **c.** The specific growth rate of strains ZQS59-ZQS65. **d.** NADH/NAD$^+$ and NADPH/NADP$^+$ ratio in strains with the synthetic energy system. ZQS39 is the control strain without the synthetic energy system, ZQS48 contains the synthetic energy system, and ZQS65 contains the optimized synthetic energy system. All data are presented as mean ± SD of biological triplicates. Statistical analysis was conducted using Student's *t*-test (two-tailed; sample size, $n = 3$). Source data are provided as a Source Data file.

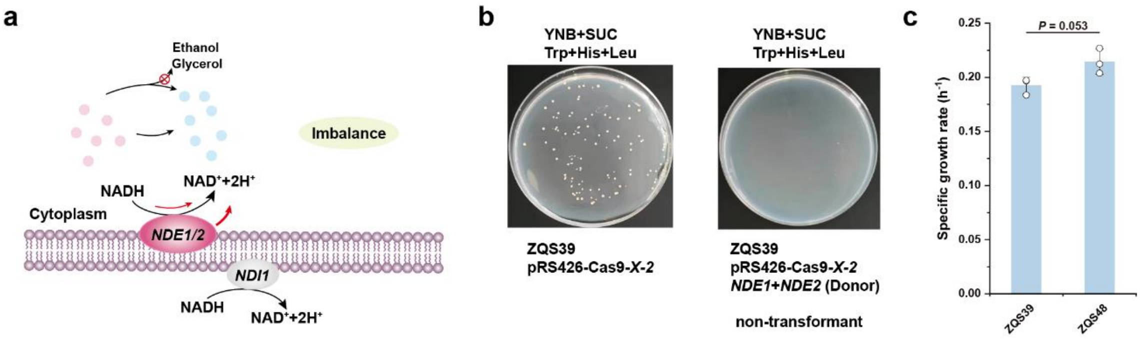

Supplementary Fig. 12. The toxic effects of *NDE1* and *NDE2* overexpression in yeast. **a.** Diagram illustrating how the overexpression of *NDE1* and *NDE2* leads to an imbalance in the cytosolic NADH/NAD$^+$ ratio. **b.** Diagram of the transformant results with *NDE1* and *NDE2* integrated into the *X-2* locus of ZQS39. The control group consisted of plates where no donor was added during the transformation, resulting in no normal transformants appeared in the experimental group. **c.** The specific growth rate of strains ZQS59-ZQS65. All data are presented as mean ± SD of biological triplicates. Statistical analysis was conducted using Student's *t*-test (two-tailed; sample size, *n* = 3). Source data are provided as a Source Data file.

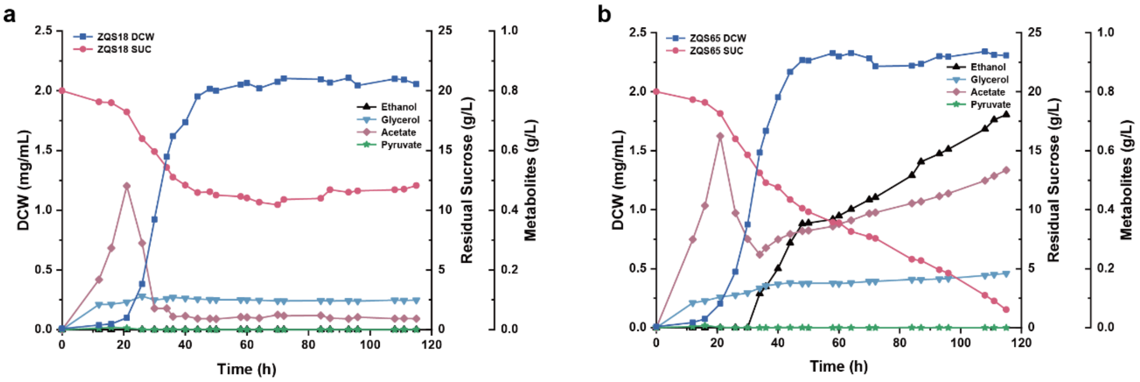

Supplementary Fig. 13. The growth status of ZQS65 and ZQS18 in a 5L-fermenter. **a.** Dynamic profiles of DCW (cell dry weight), residual sucrose, and metabolite production during ZQS18 fermentation in a 5-L bioreactor. **b.** Dynamic profiles of DCW, residual sucrose, and metabolite production during ZQS65 fermentation in a 5-L bioreactor. Source data are provided as a Source Data file.

Supplementary Table. 1. Physiological parameters of strains ZQS18 and ZQS65 during fermentation.

Strain	DCW (mg/mL)	μ_{max} (h^{-1})	Biomass yield (g DCW/g SUC)	Sucrose uptake rate (g/g DCW/h)	Ethanol (g/L)	Glycerol (g/L)	Acetate (g/L)	Pyruvate (g/L)
ZQS18	2.10	0.22	0.24	0.16	0.00	0.11	0.48	0.0076
ZQS65	2.34	0.24	0.24	0.17	0.72	0.18	0.65	0.0074
