# Supplementary material for: Sucrose-driven carbon redox rebalancing eliminates the Crabtree effect and boosts energy metabolism in yeast
Source: Nat Commun. 2025 Jun 5;16:5211. doi: 10.1038/s41467-025-60578-8 (PMC12141580; doi:10.1038/s41467-025-60578-8)
Supplement: Supplementary file 3 — Description of Additional Supplementary Files [file 41467_2025_60578_MOESM3_ESM.pdf]

Description of Additional Supplementary Files

File Name: Supplementary Data 1
Description: Summary of strategies for Crabtree effect

File Name: Supplementary Data 2
Description: All derived strains used in this study

File Name: Supplementary Data 3
Description: Detailed information about primers

File Name: Supplementary Data 4
Description: Detailed information about genes

File Name: Supplementary Data 5
Description: The plasmids used in this study

File Name: Supplementary Data 6
Description: Detailed information about gRNAs
