# Supplementary material for: Sucrose-driven carbon redox rebalancing eliminates the Crabtree effect and boosts energy metabolism in yeast
Source: Nat Commun. 2025 Jun 5;16:5211. doi: 10.1038/s41467-025-60578-8 (PMC12141580; doi:10.1038/s41467-025-60578-8)
Supplement: Supplementary file 10 — Reporting Summary [file 41467_2025_60578_MOESM10_ESM.pdf]

nature portfolio

Corresponding author(s): Yang Shan, Juan Liu

Last updated by author(s): May 20, 2025

Reporting Summary

Nature Portfolio wishes to improve the reproducibility of the work that we publish. This form provides structure for consistency and transparency in reporting. For further information on Nature Portfolio policies, see our Editorial Policies and the Editorial Policy Checklist.

Statistics

For all statistical analyses, confirm that the following items are present in the figure legend, table legend, main text, or Methods section.

n/a	Confirmed	
☐	☒	The exact sample size (*n*) for each experimental group/condition, given as a discrete number and unit of measurement
☐	☒	A statement on whether measurements were taken from distinct samples or whether the same sample was measured repeatedly
☐	☒	The statistical test(s) used AND whether they are one- or two-sided<br>*Only common tests should be described solely by name; describe more complex techniques in the Methods section.*
☒	☐	A description of all covariates tested
☒	☐	A description of any assumptions or corrections, such as tests of normality and adjustment for multiple comparisons
☐	☒	A full description of the statistical parameters including central tendency (e.g. means) or other basic estimates (e.g. regression coefficient) AND variation (e.g. standard deviation) or associated estimates of uncertainty (e.g. confidence intervals)
☐	☒	For null hypothesis testing, the test statistic (e.g. *F*, *t*, *r*) with confidence intervals, effect sizes, degrees of freedom and *P* value noted<br>*Give P values as exact values whenever suitable.*
☒	☐	For Bayesian analysis, information on the choice of priors and Markov chain Monte Carlo settings
☒	☐	For hierarchical and complex designs, identification of the appropriate level for tests and full reporting of outcomes
☒	☐	Estimates of effect sizes (e.g. Cohen's *d*, Pearson's *r*), indicating how they were calculated

Our web collection on statistics for biologists contains articles on many of the points above.

Software and code

Policy information about availability of computer code

Data collection	Shimadzu Prominence LC-20A high-performance liquid chromatography; Shimadzu SPD-20A UV-VIS detector; Shimadzu RID-10A differential refractive index detector; and BioTek Synergy H1 microplate reader.
Data analysis	Shimadzu LabSolutions 5.91 software for HPLC data analysis; Oringin 2019 64 bit for data analysis; SPSS Statistitcs 26 for statistical analysis; GrowthRates package for lag phase time calculation; and the Biocloud online tool for transcriptome data analysis.

For manuscripts utilizing custom algorithms or software that are central to the research but not yet described in published literature, software must be made available to editors and reviewers. We strongly encourage code deposition in a community repository (e.g. GitHub). See the Nature Portfolio guidelines for submitting code & software for further information.

Data

Policy information about availability of data

All manuscripts must include a data availability statement. This statement should provide the following information, where applicable:

- Accession codes, unique identifiers, or web links for publicly available datasets
- A description of any restrictions on data availability
- For clinical datasets or third party data, please ensure that the statement adheres to our policy

Raw data of RNA-seq is deposited in National Center for Biotechnology Information (NCBI) with accession numbers PRJNA1182066 (https://www.ncbi.nlm.nih.gov/bioproject/1182066). Strains, primers, genes, plasmids, and gRNA used in this work are provided in Supplementary Table 1-5. The data supporting the findings of this work are available within the paper and the Supplementary Information file. Source Data are provided with this paper.

Research involving human participants, their data, or biological material

Policy information about studies with human participants or human data. See also policy information about sex, gender (identity/presentation), and sexual orientation and race, ethnicity and racism.

Reporting on sex and gender	N/A
Reporting on race, ethnicity, or other socially relevant groupings	N/A
Population characteristics	N/A
Recruitment	N/A
Ethics oversight	N/A

Note that full information on the approval of the study protocol must also be provided in the manuscript.

Field-specific reporting

Please select the one below that is the best fit for your research. If you are not sure, read the appropriate sections before making your selection.

☒ Life sciences ☐ Behavioural & social sciences ☐ Ecological, evolutionary & environmental sciences

For a reference copy of the document with all sections, see nature.com/documents/nr-reporting-summary-flat.pdf

Life sciences study design

All studies must disclose on these points even when the disclosure is negative.

Sample size	Generally, three independent samples were adopted for data analysis due to relatively small deviation of microbial cells, which is alsostandard in metabolic engineering field that is sufficient. For some special experiments, more, or fewer sample sizes are adopted, as stated in the figure legends.
Data exclusions	No data exclusion in this study.
Replication	All the biochemical and biological experiments were performed in three replications or more. All attempts at replication were successful.
Randomization	This is not relevant to our study. Generally, the cultivation of microbial cells dose not require allocated experimental groups. In our study, yeast colonies from transformations were randomly selected for further analysis.
Blinding	No blinding was used in this study. Blinding is not applicable to metabolic engineering experiments.

Reporting for specific materials, systems and methods

We require information from authors about some types of materials, experimental systems and methods used in many studies. Here, indicate whether each material, system or method listed is relevant to your study. If you are not sure if a list item applies to your research, read the appropriate section before selecting a response.

Materials & experimental systems

n/a	Involved in the study
☒	☐ Antibodies
☒	☐ Eukaryotic cell lines
☒	☐ Palaeontology and archaeology
☒	☐ Animals and other organisms
☒	☐ Clinical data
☒	☐ Dual use research of concern
☒	☐ Plants

Methods

n/a	Involved in the study
☒	☐ ChIP-seq
☒	☐ Flow cytometry
☒	☐ MRI-based neuroimaging

Plants

Seed stocks	N/A
Novel plant genotypes	N/A
Authentication	N/A
